# Origin of asymmetric broadening of Raman peak profiles in Si nanocrystals

**DOI:** 10.1038/srep43602

**Published:** 2017-02-27

**Authors:** Yukun Gao, Penggang Yin

**Affiliations:** 1 Key Laboratory of Bio-inspired Smart Interfacial Science and Technology of Ministry of Education, School of Chemistry and Environment, Beihang University, Beijing 100191, China

## Abstract

The asymmetric peak broadening towards the low-frequency side of the Raman-active mode of Si nanocrystals with the decreasing size has been extensively reported in the literatures. In this study, an atomic coordination model is developed to study the origin of the ubiquitous asymmetric peak on the optical phonon fundamental in the Raman spectra of Si nanocrystals. Our calculation results accurately replicate the line shape of the experimentally measured optical Raman curves. More importantly, it is revealed that the observed asymmetric broadening is mainly caused by the surface bond contraction and the quantum confinement.

Raman spectra signals on crystals display superior sensitivity to subtle changes of crystal structure and composition. Some crystal defects are not easily observed by XRD and TEM, but they are clearly identified detecting by Raman spectrometer^1^,^2^,^3^,^4^. An asymmetric trend of the main Raman peak of Si nanocrystals with decreasing nanocrystal size was observed^5^. The asymmetry and broadening around the main Raman peak in symmetric stretching vibration region undoubtedly correspond to the variant of secondary-structure, which is usually considered as a surface effect of nanocrystals, and assigned as a “surface optical” phonon.

In Richter-Wang-Ley (RWL) theory^6^, the Raman intensity is given by


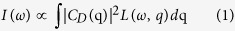


where *C*_D_(q) is the Fourier coefficients of an envelope function, and D is the nanocrystal diameter. *L(ω, q*)is Lorentzian response associated to each Raman active phonon:


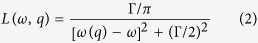


where *ω(q*) is the bulk dispersion, and Γ is the intrinsic linewidth. For microcrystalline silicon films, the broadening of the Raman peaks calculated from Eq. 2 of the dispersion relation in bulk Si is in good agreement with experimental data^6^. However, for nanocrystals with small size, the asymmetric and broadening of Raman spectra cannot be described using the model^5^. More sophisticated techniques, especially improving a weighting function and a dispersion curve should be used to perfectly fit the experimental results, although some of the functions has no physical significance^7^,^8^,^9^,^10^,^11^. Khoo *et al*. have attributed this asymmetric broadening to the increased proportion of under-coordinated atoms presented at the nanocrystal surface and the bond length contraction^12^. However, they only examined confinement effects on the Raman spectra of Si nanocrystals smaller than 3 nm due to computational constraints. Recently, the effect of surface atoms on the redshift of Raman spectra of nanocrystals has been revealed by our group^13^. In this work, we employed this atomic coordination model to study the role of silicon atoms with different coordination in asymmetric broadening of Si nanocrystals’ Raman peak.

We considered to analyze the Raman spectra of Si nanocrystals by decomposing Si nanocrystal into four type of Si atoms: one coordinated Si atoms (denoted as Si ^(1)^), two coordinated Si atoms (denoted as Si ^(2)^), three coordinated Si atoms (denoted as Si ^(3)^) and four coordinated Si atoms (denoted as Si ^(4)^). Si ^(1)^, Si ^(2)^, Si ^(3)^ represent atoms at the surface region of the nanocrystals while Si ^(4)^ represents atoms inside the nanocrystallite core. Each type of Si atoms can produce a Raman subpeak. The Raman spectrum of Si nanocrystals is a sum of four broadened symmetric Lorentzian subpeaks of Si atoms with different coordination. The ratio of the relative integrated intensity between each two different coordinated Si atoms is proportional to their number of atoms.

According to the theory^13^, we could determine the frequency positions (*ω(D*)^(*i*)^) of Si^(*i*)^ atoms (*i* = 1, 2, 3, 4) by using the function *ω(D*)^(*i*)^ = *ω(∞*) − Δ*ω(D*) ^(*i*)^, where the Raman shifts (Δ*ω(D*) ^(*i*)^) of Si^(*i*)^ atoms can be expressed as


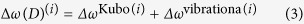


where *Δω*^Kubo(*i*)^, *Δω*^vibrationa(*i*)^ are the quantum effect shift and the vibrational shift, respectively.


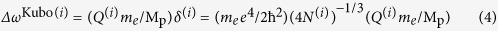


where *Q*^(*i*)^ = 2(*Z*_c_/*N*_c_^(*i*)^), *Z*_c_ and *N*_c_^(*i*)^ are the valence and coordination of the Si^(*i*)^ atom. *N*^(*i*)^ is the number of the Si^(*i*)^ atom.


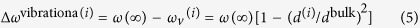


*ω*_v_^(*i*)^ and *ω(∞*) represent the vibration frequency of the Si^(*i*)^ atoms and the bulk phase, respectively. *d*^(*i*)^ and *d*^bulk^ represent the bond lengths of the Si^(*i*)^ atoms and the bulk phase, respectively.

Therefore, The Raman frequency position *ω(D*)^(*i*)^ of Si^(*i*)^ atoms can be expressed as


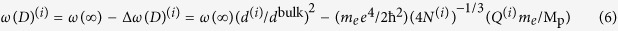


From Eq. 6, it can be seen that Raman peak positions of Si^(1)^, Si^(2)^, Si^(3)^ are due to the competing influences of bond length contraction and number of atoms of Si, which is in agreement with Khoo’s first principles results^12^. For Si^(4)^ atoms, the Raman frequency position is only determined to Kubo confinement effect due to *d*^(4)^ = *d*^bulk^.

In ref. 5, Raman spectra for Si nanocrystals with size of 2.7 to 11.8 nm are reported. The measured Raman spectra of the Si nanocrystals in ref. 5 are plotted as black open squares in Fig. 1. It can be seen that the Raman-active mode of Si is accompanied by asymmetric peak broadening with the decreasing size. This asymmetric vibration mode is exceedingly significant for the smaller Si nanocrystals with size of 2.7 and 2.9 nm. Hessel pointed out that the RWL model could not capture the proper line shape of the low-frequency tail of the Raman peaks^5^. In order to match the observed peak shape, besides the RWL model curve, an additional Lorentzian feature was in need. In Figure S2 of ref. 5, the spectra were fit using symmetric Lorentzians with arbitrary peak position. For contrast, symmetric Lorentzians with certain peak position of Si^(*i*)^ atoms are employed to fit the Raman spectrum in our work. Raman frequency positions of the Si^(*i*)^ atoms can be predicted by Eq. 6, with results listed in Table 1. The relative integrated intensity of Lorentzians peaks of Si^(*i*)^ atoms are determined by the number of Si^(*i*)^ atoms, reflected from Table 2. It can be seen that the peak position of spectral band of Si^(*1*)^ atoms is far from the core position of Raman spectra. Since the number of Si^(*1*)^ are very small, relative integrated intensities of spectral band of Si^(*1*)^ atoms is also so small that may be ignored. Ultimately, the measured Raman spectra of Si nanocrystals can be satisfactorily fitted with three Lorentzianwhose peak positions determined by using Eq. 6, shown in Fig. 1. The yellow, magenta, and cyan curve correspond to a symmetric Lorentzian of Si^(2)^, Si^(3)^, and Si^(4)^ atoms, respectively. Blue solid curve is the sum of yellow, magenta, and cyan curve. The subpeak of Si^(4)^ is only due to confinement effect while that of Si^(2)^ and Si^(3)^ is due to combination of the confinement and bond contraction effect. It is noticed that the large Lorentzian peak of Si^(4)^ atoms is slightly red-shifted and broadened as the nanocrystal size become smaller.

From Table 2, we find that the ratio of the integrated intensity, I(Si^(3)^)/I(Si^(4)^) and I(Si^(2)^)/I(Si^(4)^), show obvious crystallite-size dependence. The relative intensity ratios show an almost proportional relationship with increasing size in the range of 2.9–11.8 nm. When the crystallite size increases up to 11.8 nm, the intensity of spectral peak of Si^(2)^ atoms almost diminishes. For 2.9 nm Si nanocrystal, the intensity ratios of the I(Si^(3)^)/I(Si^(4)^) and I(Si^(2)^)/I(Si^(4)^) is about 0.25 and 0.22, respectively. As a result, the ratios rapidly decrease with increasing crystallite size. This change of the intensity ratio results from the number of atoms inside the nanocrystal becomes greater than that of the surface atoms with the nanocrystals’ increasing size.

In summary, we have studied the vibration and Raman spectra of Si nanocrystals based on an atom coordination model. We find that the measured Raman spectra of Si nanocrystals can be well reproduced by a sum of four broadened symmetric Lorentzian subpeaks of Si atoms with different coordination. Moreover, the ratio of the relative integrated intensity of symmetric Lorentzian subpeaks between different coordinated Si atoms presents proportional to their number of atoms in our study.

## Methods Section

According to the method in ref. 13, the number of surface atoms and the length of bond at the surface of the semiconductor nanocrystals can be obtained using the Material studio program of Accelrys Inc. The geometry optimization by first principles calculations was carried out using the GGA approach by the Perdew-Burke-Ernzerhof (PBE)^14^ exchange correlation functional of density functional theory with the Material studio^15^. The calculations were performed using an energy cutoff of 380 eV with the plane wave basis set. A 4 × 4 × 4 k-point Monkhorst-Pack grid is used.

## Additional Information

**How to cite this article**: Gao, Y. and Yin, P. Origin of asymmetric broadening of Raman peak profiles in Si nanocrystals. *Sci. Rep.* **7**, 43602; doi: 10.1038/srep43602 (2017).

**Publisher's note:** Springer Nature remains neutral with regard to jurisdictional claims in published maps and institutional affiliations.

## Figures and Tables

**Figure 1:**
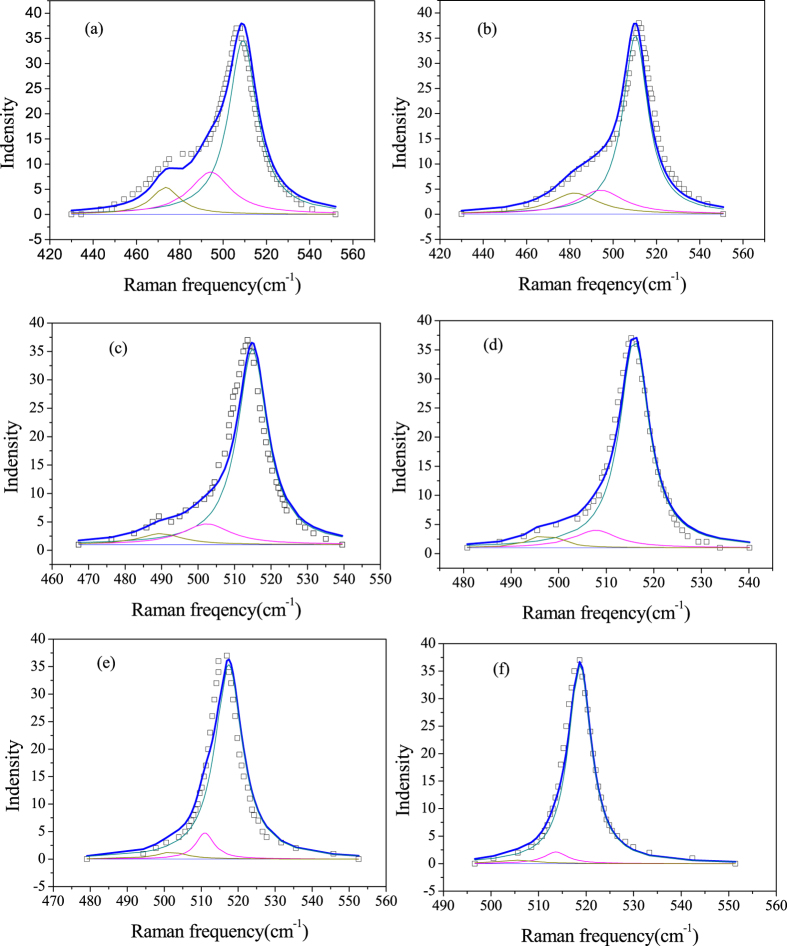
Measured (black open squares reproduced from ref. 5) and calculated Raman spectra of Si nanocrystals with size of 2.7 nm (**a**), 2.9 nm (**b**), 5.0 nm (**c**), 6.0 nm (**d**), 8.8 nm (**e**) and 11.8 nm (**f**). The spectra were fit to the model of this work (blue curve). The yellow, magenta, and cyan curve correspond to a symmetric Lorentzian with certain peak position of Si (2), Si (3), and Si (4) atoms, respectively. Blue curve is a sum of yellow, magenta, and cyan curve.

**Table 1:** Calculated Raman peak positions *ω(D*)^(i)^ of Lorentzian peaks of Si^(*i*)^ atoms.

Size (nm)	*ω(D*)^(1)^	*ω(D*)^(2)^	*ω(D*)^(3)^	*ω(D*)^(4)^
2.7	409.0	473.4	494.2	509.1
2.9		482.1	494.2	510.3
5.0	381.3	489.9	502.4	514.9
6.0	431.1	493.1	503.6	515.9
8.8	431.1	497.6	506.7	517.4
11.8	449.4	501.0	509.2	518.7

**Table 2:** Number of atoms and relative integrated intensities of Lorentzian peaks of Si^(*i*)^ atoms.

Size (nm)	Number of Si^(1)^	Number of Si^(2)^	Number of Si^(3)^	Number of Si^(4)^	I(Si^(2)^)/I(Si^(4)^)	I(Si^(3)^)/I(Si^(4)^)
2.7	24	48	108	309	0.16	0.35
2.9	0	96	108	429	0.22	0.25
5.0	12	210	448	2632	0.08	0.17
6.0	48	312	604	4743	0.07	0.13
8.8	48	612	1432	15792	0.04	0.09
11.8	100	1140	2520	39229	0.03	0.06
